# The Persistence-Inducing Toxin HokB Forms Dynamic Pores That Cause ATP Leakage

**DOI:** 10.1128/mBio.00744-18

**Published:** 2018-08-14

**Authors:** Dorien Wilmaerts, Mariam Bayoumi, Liselot Dewachter, Wouter Knapen, Jacek T. Mika, Johan Hofkens, Peter Dedecker, Giovanni Maglia, Natalie Verstraeten, Jan Michiels

**Affiliations:** a Center for Microbiology, VIB, Leuven, Belgium; b Centre of Microbial and Plant Genetics, KU Leuven, Leuven, Belgium; c Biochemistry, Molecular and Structural Biology Section, KU Leuven, Leuven, Belgium; d Molecular Imaging and Photonics, KU Leuven, Leuven, Belgium; e Groningen Biomolecular Sciences & Biotechnology (GBB) Institute, University of Groningen, Groningen, The Netherlands; National Institute of Child Health and Human Development (NICHD)

**Keywords:** persistence, pore-forming peptide, toxin-antitoxin modules

## Abstract

Bacterial populations harbor a small fraction of cells that display transient multidrug tolerance. These so-called persister cells are extremely difficult to eradicate and contribute to the recalcitrance of chronic infections. Several signaling pathways leading to persistence have been identified. However, it is poorly understood how the effectors of these pathways function at the molecular level. In a previous study, we reported that the conserved GTPase Obg induces persistence in Escherichia coli via transcriptional upregulation of the toxin HokB. In the present study, we demonstrate that HokB inserts in the cytoplasmic membrane where it forms pores. The pore-forming capacity of the HokB peptide is demonstrated by *in vitro* conductance measurements on synthetic and natural lipid bilayers, revealing an asymmetrical conductance profile. Pore formation is directly linked to persistence and results in leakage of intracellular ATP. HokB-induced persistence is strongly impeded in the presence of a channel blocker, thereby providing a direct link between pore functioning and persistence. Furthermore, the activity of HokB pores is sensitive to the membrane potential. This sensitivity presumably results from the formation of either intermediate or mature pore types depending on the membrane potential. Taken together, these results provide a detailed view on the mechanistic basis of persister formation through the effector HokB.

## IMPORTANCE

There is increasing awareness of the clinical importance of persistence. Indeed, persistence is linked to the recalcitrance of chronic infections, and evidence is accumulating that persister cells constitute a pool of viable cells from which resistant mutants can emerge. Unfortunately, persistence is a poorly understood process at the mechanistic level. In this study, we unraveled the pore-forming activity of HokB in E. coli and discovered that these pores lead to leakage of intracellular ATP, which is correlated with the induction of persistence. Moreover, we established a link between persistence and pore activity, as the number of HokB-induced persister cells was strongly reduced using a channel blocker. The latter opens opportunities to reduce the number of persister cells in a clinical setting.

## INTRODUCTION

Persister cells are rare, phenotypic variants in isogenic bacterial populations that are transiently dormant, thereby surviving treatment with high doses of bactericidal antibiotics. Following antibiotic removal, persister cells regrow and form a new population, which is again sensitive to the antibiotic (1). Persisters contribute to the recalcitrance of chronic infections, and recent findings suggest that they constitute a pool from which resistant mutants can emerge (2–4). Multiple pathways may lead to the formation of persisters, making their study challenging (1).

Toxin-antitoxin (TA) modules are important mediators of persistence (5–10), although the detailed mode of action of these effectors is often unknown (11). TA modules are located on plasmids and chromosomes and encode a stable toxin, targeting an essential cellular function, and an unstable antitoxin that inhibits its cognate toxin. While plasmid-encoded TA modules contribute to plasmid maintenance via a mechanism called postsegregational killing (12, 13), genomic TA modules play a role in survival in stressful and fluctuating environments (14). In the latter case, the antitoxin is degraded through stochastic variation or as a result of stress, resulting in toxin activation (15). When their concentration exceeds a certain threshold, toxins can inhibit an essential cellular function, which in some cases induces persistence (16).

TA modules are classified into six types, depending on the mode of action of the antitoxin. Type I antitoxins are small regulatory RNAs that base pair to their cognate toxin mRNA, thereby inhibiting translation and in some cases triggering degradation of toxin mRNA (13). The majority of the characterized type I toxins are assumed to be membrane localized, and this has been confirmed biochemically for a limited set of toxins (17). However, direct microscopic visualization of the membrane localization in cells has not been established. In addition, expression of several type I toxins has been shown to collapse the membrane potential, indicative of pore formation (17). However, this pore-forming activity has been experimentally verified only for TisB (18). Overall, a detailed mode of action of type I toxins is often lacking. Work from our group revealed that in Escherichia coli, expression of the conserved GTPase Obg induces persistence through transcriptional activation of the type I toxin HokB. While deleting *hokB* does not influence persistence, overexpressing *hokB* increases the number of persister cells (9). HokB consists of 49 amino acids and is a member of the Hok/Gef family, consisting of small, toxic proteins that are widespread in Gram-negative bacteria. However, the working mechanism of HokB and the relation with persistence in E. coli is unknown.

In the present study, we combine *in vitro* and *in vivo* experiments to unravel the mode of action of HokB in E. coli persistence. Using planar lipid bilayers, we show that HokB forms pores with nonlinear current-voltage behavior. We demonstrate that the channel blocker polyethylene glycol (PEG) reduces *hokB*-induced persistence, indicative of a direct link between pore formation and persistence. HokB pores lower the cellular energy status (ATP/ADP ratio) of the cells and cause leakage of intracellular ATP, thereby inducing dormancy.

## RESULTS

### HokB forms pores.

**FIG 1:**
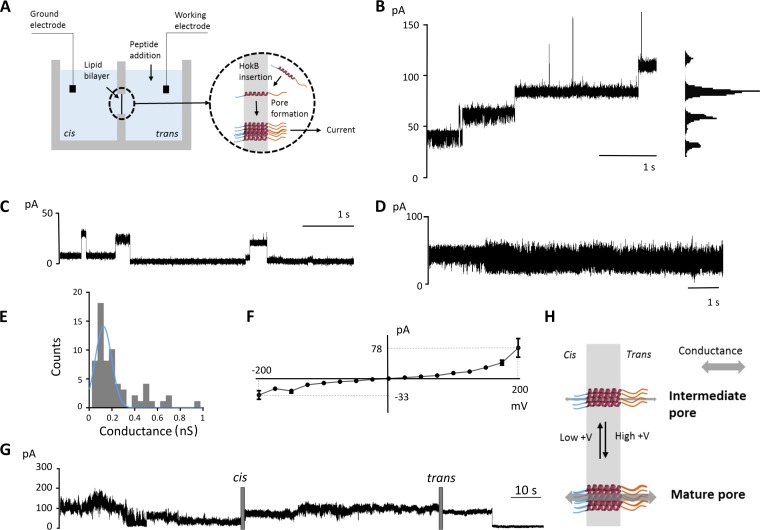
(A) Schematic representation of the setup of the conductance measurements on planar lipid bilayers. Peptides are added in *trans*, at the side of the working electrode. A high voltage is applied to force HokB insertion in the lipid bilayer. After insertion, a pore is formed providing a current when a voltage is applied. The transmembrane part of HokB is shown as an α-helix, with the N-terminal domain (blue) and the C-terminal domain (orange) indicated. (B) Trace showing a stepwise increase in the current visualized in the histogram (+200 mV). (C) Trace showing that HokB is able to form a pore with multiple conductances (+200 mV). (D) Trace of HokB-induced pores showing a noisier outcome (+100 mV). (E) Histogram of conductance measurements of 63 independent single-step insertions of HokB peptides in a bilayer of DPhPC lipids. A Gaussian curve (blue curve) was fitted to the data to calculate the most abundant conductance (0.12 ± 0.01 nS). (F) Current-voltage relation for HokB-induced single pores using DPhPC lipids. Voltage was constant for 1 s and varied between +200 mV and −200 mV, in steps of 25 mV. The averages ± standard errors of the means (SEM) (error bars) of eight independent pores are presented. (G) Upon pore formation, addition of mPEG5k-mal at the *cis* side does not influence ion current (99.74 pA), while sequential addition to the *trans* side strongly reduces ion flow (9.78 pA) (+100 mV). (H) An applied high positive voltage induces a strong (nonlinear) increase in conductance, presumably through the formation of a mature pore, while intermediate pores are predominantly present when a lower positive voltage is applied.

Planar lipid bilayers are often used to assess ion channel activities (19) or the behavior of pore-forming peptides (18, 20). We used this technique to assess pore formation by HokB in a bilayer formed with the synthetic lipid diphytanoylphosphatidylcholine (DPhPC). HokB peptides were added at one side (*trans*, working electrode) of the planar lipid membranes to mimic the asymmetry of the *in vivo* membrane. A voltage (up to ±200 mV) was applied to force insertion of peptides into the planar lipid membrane, as spontaneous insertion was inefficient. A schematic representation of the setup is depicted in Fig. 1A. Upon peptide addition, an increase in membrane conductance was observed, indicating that membrane-inserted HokB peptides form pores. A high positive voltage (approximately +200 mV) was often necessary for pore activation. Upon pore formation, three different behaviors could be observed. First, a stepwise increase in current (Fig. 1B), overall at voltages of >100 mV, was detected. This could result from different conductance states of one pore or from the subsequent activation of different pores. Second, some pores showed multiple conductances in combination with opening and closing of the pore (Fig. 1C). Pores were open for 5.90 ± 2.11 s (mean ± standard error of the mean [SEM]) and closed for 6.00 ± 2.37 s. Third, in 10% of the cases, the observed pore displayed a noisier outcome (Fig. 1D).

To further characterize HokB-induced pores in the synthetic lipid bilayer, membrane insertions were examined at different holding voltages. For approximately 80% of the pores, applying a voltage with positive polarity to the *trans* compartment resulted in a higher conductance than applying a negative polarity. For the remaining 20% of pores, the conductance was independent of the sign of the applied voltage, possibly because breaking and reforming the bilayer results in contamination of the peptide to the *cis* side. To estimate the single-pore conductance, frequency distributions of the measured conductances at +100 mV were determined (Fig. 1E). The most abundant conductance (0.12 nS), which is an estimate for the single-pore conductance, was used to calculate a pore diameter of 0.59 nm using the model of Hille.

To mimic a more relevant environment for the peptide, conductance measurements were performed with extracted E. coli lipids, deprived of the lipopolysaccharide (LPS) layer (see Fig. S1 in the supplemental material). As was the case with DPhPC lipids, the conductance of HokB pores was asymmetrical and depended on the polarity of the applied voltage. Pore conductance was larger when applying a voltage with negative polarity (for 49% of the observed pores), positive polarity (for 43% of the observed pores), or independent of the sign of the applied voltage (for 8% of the observed pores). These differences in activating potentials are expected, as natural lipids result in a less dense bilayer due to the presence of cardiolipin (21), which may allow peptide insertion in different orientations. The activating potentials (+100 mV or −100 mV) were used to calculate the frequency distribution of the conductances, showing that the most abundant conductance of pores formed in natural lipid membranes (0.14 nS) is similar to the most abundant conductance found using the DPhPC lipids (0.12 nS). Using the model of Hille, the diameter of the HokB pores in the natural lipid bilayer was estimated to be 0.64 nm (22).

10.1128/mBio.00744-18.1 FIG S1  Histogram of conductance measurements of 35 independent single-step insertions of HokB peptides in the bilayer using extracted E. coli lipids. A Gaussian curve was fitted to the data to calculate the most abundant conductance (0.14 ± 0.097 nS [mean ± standard deviation {SD}]). Download FIG S1, TIF file, 0.04 MB. Copyright © 2018 Wilmaerts et al. 2018 Wilmaerts et al. This content is distributed under the terms of the Creative Commons Attribution 4.0 International license.

### Peptide insertion is asymmetrical.

Current-voltage (I-V) measurements obtained from single-pore conductances using the synthetic lipids show asymmetrical behavior (Fig. 1F). Although conductance increases nonlinearly at both increasing positive and negative potentials, it is clear that applying a potential with positive polarity to the *trans* compartment results in higher pore currents than applying a negative polarity. This asymmetrical behavior is called ionic current rectification. The rectification ratio (α) is scored by dividing the conductance at negative potential over the conductance at positive potential (23), giving an α of 0.42 for HokB peptides at +200 mV. As this asymmetrical I-V curve is an indication of orientational rather than random insertion into the lipid bilayer, we assessed the orientation of HokB pores.

As HokB belongs to the Hok/Gef family, it is predicted to be a single-span membrane peptide (24). Also, computational predictions using the Kyte-Doolittle algorithm and the CBS TMHMM tool (25, 26) show that HokB is a single-span membrane peptide with a long, negatively charged C-terminal periplasmic tail and a short, positively charged N terminus extending in the cytoplasm (Fig. S2). Presumably, these positive charges, conserved in all Hok peptides studied thus far, anchor the N-terminal part in the cytoplasm (27, 28). HokB contains three cysteine residues (C9, C14, and C46). C9 and C14 are predicted to be part of an α-helix and are buried in the lipid bilayer (25, 26), leaving only the C-terminal C46 residue exposed. The C46 residue, either present in *cis* or in *trans*, can react with the thiol-directed PEG reagent methoxy polyethylene glycolmaleimide (mPEG-mal), which would impede current flow (29). Consequently, the orientation of the pore can be determined by comparing the change in conductance when mPEG5k-mal is added at the *cis* side versus the *trans* side. The radius of the used mPEG5k-mal (5,000 Da) is estimated to be 2.55 nm (30), well above the estimated size of the HokB pore (0.59 nm). Therefore, addition to one side of the compartment excludes the mPEG5k-mal from the other side. For this experiment, HokB peptides were added to the *trans* side of a newly formed bilayer. The bilayer was not broken and reformed to prevent contamination of peptides in the *cis* compartment. A representative example of a trace is depicted in Fig. 1G. The pore formed showed noisy behavior, with conductances of 0.32 and 1.08 nS. After the addition of mPEG5k-mal in *cis*, conductance was unaffected (1.00 nS), indicating that there was no reaction between mPEG5k-mal and a thiol. Conversely, upon the addition of mPEG5k-mal in *trans*, conductance decreased to 0.098 nS in a single step, resulting from the reaction between mPEG5k-mal and C46. These results indicate that, upon insertion, HokB crosses the bilayer with the N terminus, as depicted in Fig. 1A. When extrapolating these *in vitro* results to the *in vivo* situation and taking the orientation of the HokB pores into account, a positive applied voltage corresponds to a polarized biological membrane.

10.1128/mBio.00744-18.2 FIG S2  TMHMM analysis (26) of the HokB peptide. The N-terminal domain is predicted to be localized in the cytoplasm (amino acids 1 to 6). From amino acids 7 to 29, there is a high probability for a transmembrane helix. The C-terminal domain is predicted to be in the periplasm (amino acids 30 to 49). Download FIG S2, TIF file, 0.2 MB. Copyright © 2018 Wilmaerts et al. 2018 Wilmaerts et al. This content is distributed under the terms of the Creative Commons Attribution 4.0 International license.

Remarkably, although conductance before the addition in *trans* (1.00 nS) was far above the calculated single-pore conductance (0.12 nS), the single-step inhibition indicates that only one pore was formed. This can be explained by the fact that HokB pores display not only asymmetrical behavior but also pronounced nonlinear behavior, with a strong increase in conductance upon applying a positive voltage in increments (Fig. 1F). This presumably proceeds via the formation of low-conductance pores (hereafter referred to as intermediate pores) at low potentials, which are maturating toward high-conductance pores (hereafter referred to as mature pores) at high potentials (Fig. 1H). In subsequent experiments, the membrane activity of HokB peptides was further examined *in vivo*.

### Pore formation and activity are important for HokB-induced persistence.

Our *in vitro* experiments demonstrate that HokB targets the lipid bilayer and forms pores. To extend these results to the *in vivo* situation, we first confirmed the cellular localization of HokB using conventional microscopy with HokB N-terminally tagged with mCherry (Fig. S3A and B) and by superresolution microscopy (photoactivated localization microscopy [PALM]) with HokB N-terminally tagged with *p*hoto*a*ctivatable (PA)-mCherry (Fig. S3C and D). Both constructs are still functional, as they induce persistence (Fig. S3E) and confirm the membrane localization of HokB. Moreover, cluster formation of HokB peptides can be observed (Fig. S3B).

10.1128/mBio.00744-18.3 FIG S3  Visualization of HokB localization in E. coli in randomly selected cells using conventional fluorescence microscopy (A and B) and PALM (C and D). Fluorescence is homogeneously distributed over the entire cell when expressing *mCherry* (A) or *PAmCherry* (C). Fluorescence is located at the cell membrane when expressing *mCherry-hokB* (B) or *PAmCherry-hokB* (D). The color scale for PALM represents the minimal and maximal fluorescence intensity of a given spot (50). (E) Expression of both *mCherry-hokB* and *PAmCherry-hokB* in E. coli K-12 BW25113 increases the persister fraction significantly. Values are means ± SEM from at least three independent experiments. Asterisks indicate statistical significance (*, *P* < 0.01; ***, *P* < 0.0001). Download FIG S3, TIF file, 0.3 MB. Copyright © 2018 Wilmaerts et al. 2018 Wilmaerts et al. This content is distributed under the terms of the Creative Commons Attribution 4.0 International license.

**FIG 2:**
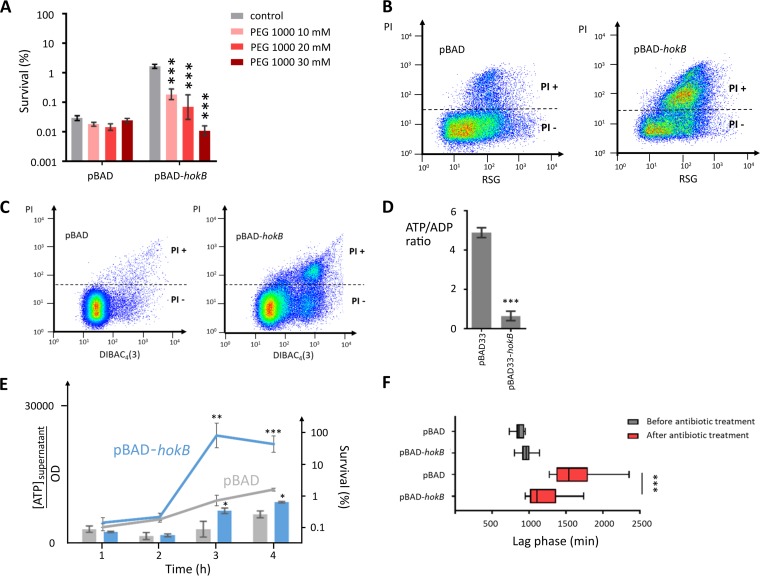
(A) Addition of high concentrations of PEG 1000 does not affect the empty vector control but abolishes *hokB*-induced persistence. Values are means ± SEM (error bars) from at least three independent experiments. Asterisks indicate statistical significance against the control (no PEG 1000 added) (***, *P* < 0.0001). (B) PI-positive (PI+) cells after *hokB* expression are not dead, as the RSG staining shows that they are metabolically active. The mean RSG values are 94.35 (±6.58) for PI-negative (PI−) cells and 174.67 (± 12.89) for PI+ cells. Values are means ± SEM from at least three experiments. A representative plot of at least three independent experiments is shown. (C) PI-positive cells after *hokB* expression are enriched in the subpopulation with a depolarized membrane, measured with DiBAC_4_(3). A representative plot of at least three independent experiments is shown. (D) *hokB* expression lowers the cellular energy ratio of stationary-phase cells. Values are means ± SEM from at least three independent experiments. Asterisks indicate statistical significance compared to the empty vector control (***, *P* < 0.0001). (E) *hokB* expression results in an increase in ATP in the supernatant from 3-h induction (left *y* axis). The release of ATP in the supernatant occurs concomitantly with the induction of persistence by *hokB* in time (right *y* axis). Values are means ± SEM from at least three independent experiments. Asterisks indicate statistical significance compared to the empty vector control (*, *P* < 0.05; **, *P* < 0.001; ***, *P* < 0.0001). The increase of ATP in the supernatant is not caused by HokB-induced cell lysis, as the concentration of proteins in the supernatant upon expressing *hokB* is constant in time and unchanged compared to a control sample (71.51 ng µl^−1^ ± 17.49 for pBAD and 63.17 ng µl^−1^ ± 17.49 for pBAD-*hokB* [values represented as means ± SEM]). (F) *hokB* expression lowers the lag before persister cell outgrowth but does not affect the untreated control. Values are means ± SEM from at least three independent experiments. Asterisks indicate significant differences between pBAD and pBAD-*hokB* (***, *P* < 0.0001).

As we hypothesized that pore formation would be important for HokB-induced persistence, we quantified persister cell levels in the presence of PEGs that can transiently occupy the open pore and impede current flow (31). These blocking events depend on the molecular weight of the PEG and therefore, given the predicted size of HokB pores (~0.59 nm), a subset of differently sized channel blockers were selected (PEG 200, PEG 400, PEG 600, and PEG 1000). Ethylene glycol and PEG 6000 were used as controls, since ethylene glycol is too small to influence pore conductance and PEG 6000 is too large to enter the pore, as the estimated Stokes radius (3.2 nm) is far above the estimated pore size of HokB (30). Results shown in Fig. S4 indicate that PEG 1000, with an estimated radius of 1 nm (30), has the strongest effect on HokB-induced persistence (14-fold). Moreover, an increase in concentration of PEG 1000 completely abolishes HokB-induced persistence (Fig. 2A). The finding that PEG 1000 affects persistence indicates that HokB pores have an estimated radius of ~1 nm. The cellular localization of PEG 1000 was further confirmed by using the fluorescein derivate PEG 1000-FITC (PEG 1000 labeled with fluorescein isothiocyanate [FITC]). We observed fluorescent patches in the membrane resulting from PEG 1000-FITC, which were colocalized (*P* < 0.05) with regions of higher red fluorescence intensity resulting from mCherry-labeled HokB (mCherry-HokB) clustering (Fig. S5A). Patch formation was not caused by the mCherry tag, as *hokB* expression also results in the presence of FITC fluorescent patches. Furthermore, strains expressing *mCherry* were not stained by PEG 1000-FITC (Fig. S5B).

10.1128/mBio.00744-18.4 FIG S4  HokB-induced persistence is strongly reduced by the use of the channel inhibitor PEG 1000. PEG molecules and ethylene glycol were added at 10 mM. Values are means ± SEM from at least three independent experiments. Asterisks indicate statistical significance (*, *P* < 0.01; ***, *P* < 0.0001). Download FIG S4, TIF file, 0.1 MB. Copyright © 2018 Wilmaerts et al. 2018 Wilmaerts et al. This content is distributed under the terms of the Creative Commons Attribution 4.0 International license.

10.1128/mBio.00744-18.5 FIG S5  (A) PEG 1000-FITC is located in fluorescent patches in the membrane. These patches are colocalized with the fluorescent patches visible after *mCherry-hokB* expression. (B) The control strain (*mCherry*) does not result in the formation of fluorescent patches. Patch formation is not caused by the mCherry tag, as *hokB* expression also results in the formation of fluorescent patches. Download FIG S5, TIF file, 0.2 MB. Copyright © 2018 Wilmaerts et al. 2018 Wilmaerts et al. This content is distributed under the terms of the Creative Commons Attribution 4.0 International license.

To further confirm the pore-forming activity of HokB, the permeabilization marker propidium iodide (PI) was used, which has a radius of 0.5 nm (32). PI cannot enter cells with intact membranes, and therefore, it is a marker for loss of membrane integrity and cell death. Upon *hokB* expression, 29.77% (±17.11% [SEM]) of the population is PI positive (PI+). This could result from the loss of membrane integrity upon *hokB* expression or from PI passage through HokB pores. To distinguish between these possibilities, we used redox sensor green (RSG), a fluorescent marker of metabolic activity that does not stain dead cells (Fig. 2B). Clearly, upon *hokB* expression, PI+ cells are not dead, since they stain with RSG. These cells even have a significantly higher metabolic activity (*P* < 0.001) compared to negative (PI−) cells. These results confirm that HokB forms pores *in vivo* through which PI can be transported.

### HokB pores mediate a decrease in cellular energy ratio and leakage of intracellular ATP.

We have previously shown that *hokB* expression lowers the electrical potential of the bacterial cell membrane (9). A direct correlation between pore activity and electrical potential, however, has not yet been established. Therefore, we simultaneously monitored at the single-cell level staining of bacteria by PI, a measure of HokB pore activity, and bis-(1,3-dibutylbarbituric acid)trimethine oxonol [DiBAC_4_(3)], a fluorescent stain that only enters depolarized cells (33). As depicted in Fig. 2C, PI+ cells are present in the subpopulation of cells with a depolarized membrane, indicative of persistence (9). Unexpectedly, we also observed a subfraction of cells that are PI− but have a depolarized membrane, presumably resulting from the formation of smaller HokB pores that still affect membrane potential but through which PI cannot enter. Because the electrical potential is an important component of the proton motive force (pmf) that drives ATP synthesis, we assessed the influence of HokB on the cellular energy ratio. To this end, we expressed *hokB* in the presence of the engineered ATP/ADP ratio sensor Perceval (34). As shown in Fig. 2D, HokB strongly reduces the cellular energy ratio.

As PEG 1000 with a radius of 1 nm was able to block HokB pores, we assessed whether intracellular ATP (~0.7 nm) could leak through the pores. Therefore, we measured ATP levels in the supernatant during induction of *hokB* (Fig. 2E). We clearly observed a strong increase in the extracellular ATP concentration 3 h after *hokB* induction, indicating that HokB pores directly transport ATP. To correlate the increased concentration of ATP in the supernatant with the induction of persistence, the number of persister cells was assessed during induction of HokB (Fig. 2E). Indeed, HokB increases the persister fraction only after 3-h induction, which correlates well with the increase in ATP concentration in the supernatant (Fig. 2E).

### Fast awakening of HokB-induced persister cells.

After antibiotic removal, persister cells can resume growth when they exit from the dormant state (1). Given the fact that HokB pores are formed in metabolically active cells and that they are relatively large and allow passage of metabolites, including ATP, we investigated whether the increased metabolic activity affected persister awakening. To examine the resuscitation of HokB-induced persister cells, single-cell lag times of persister cell outgrowth were measured. Persister cells, obtained either with or without *hokB* induction, were grown in lysogeny broth (LB), and single-cell lag times were calculated. Remarkably, the lag time of HokB-induced persister cells is significantly shorter compared to the control cells. On the other hand, expression of *hokB* does not affect the lag time of nonpersister cells (Fig. 2F). Taken together with the observation of high RSG activity, these results suggest that *hokB*-induced persister cells originate from cells with higher metabolic activity, allowing them to resume cell division faster.

## DISCUSSION

*In vitro* conductance measurements with planar lipid bilayers demonstrated pore formation by the HokB peptide. Using the model of Hille (22), we estimated the diameter of HokB pores to be 0.59 nm (synthetic lipids) and 0.64 nm (natural lipids). HokB pores are therefore larger than pores formed by TisB, another type I toxin involved in persistence, with a calculated diameter of ~0.15 nm (18).

The current-voltage (I-V) curve revealed an asymmetrical behavior regarding the polarity of the applied voltage, presumably resulting from the intrinsic asymmetry of the HokB peptide. Indeed, although the peptide is overall uncharged, the N terminus is positively charged, while the C terminus is negatively charged (20). We experimentally defined the HokB pore orientation *in vitro* using specific PEGylation (29) and demonstrated that HokB peptides cross the lipid bilayer with the N-terminal domain. This is expected, as the N-terminal domain is much shorter than the C-terminal domain, leading to a difference in the free energy barrier between the two domains (35). Because HokB insertion in a lipid bilayer is directional, the exact orientation of the peptides *in vitro* can now quickly be deduced from the asymmetrical behavior of a single-pore I-V curve.

The nonlinearity of the I-V curve does not result from the formation of multiple pores, as the 100 mV step in the I-V curve has a single-pore conductance. Furthermore, conductance higher than the conductance for a single pore was impeded by mPEG5k-mal in a single step, indicative of the presence of a single pore. Indeed, in the case of multiple pores, a multistep decrease would be expected. Therefore, a high positive voltage induces the formation of mature pores, while intermediate pores are formed at lower applied voltages. With the experimentally defined orientation of HokB pores, a positive potential mimics a cell with a polarized membrane, indicating that mature HokB pores are formed in cells with a high membrane potential.

In addition to a stepwise increase in current, most likely resulting from different intermediate steps in HokB pore formation, HokB pores display two other types of behavior. First, some HokB pores show multiple conductances with consecutive opening and closing of the pore, thereby resembling voltage-independent gating-like behavior. Second, some HokB pores show more noisy behavior. Whether these two HokB characteristics play a role in the formation and awakening of HokB-induced persister cells is currently unclear.

Using channel blockers, we were able to assess HokB pore formation and probe pore size *in vivo*. Upon addition of PEG 1000 at high concentration, *hokB*-induced persister formation was completely abolished, indicating that pores *in vivo* (~2-nm radius) are bigger than pores *in vitro*. As clusters of PEG 1000-FITC colocalized with clusters of the HokB peptides, the effect of PEG 1000 on persistence most likely results from blocking of the pore, although other effects cannot be excluded. Moreover, cells stained with PI resulting from *hokB* expression have a strongly depolarized membrane, which is linked with persistence (9). Taken together, these results provide the first direct link between pore activity of a type I toxin and persistence.

Pore formation results in membrane depolarization, a decrease in cellular energy ratio, and leakage of intracellular ATP. This leakage occurs concomitantly with the emergence of HokB-induced persister cells, indicating that it may be involved in the induction of persistence. The link between cellular energy levels and persistence confirms previous results from other groups demonstrating that persister formation is correlated with ATP depletion, as observed in Staphylococcus aureus (36) and E. coli (37).

**FIG 3:**
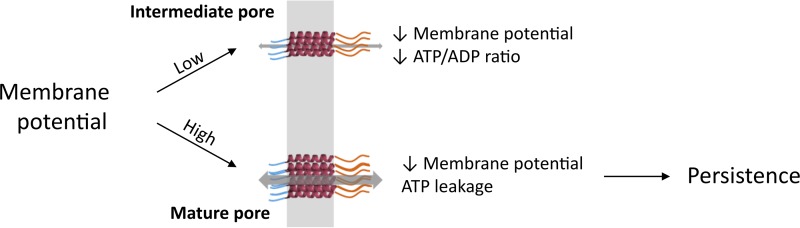
Model of dynamic HokB pore formation. Mature pores are formed when the membrane potential is high, resulting in a decrease in membrane potential and leakage of intracellular ATP. Together, this will induce persistence. Intermediate pores are formed when the membrane potential is low. They collapse the membrane potential, thereby decreasing the cellular energy ratio.

Upon *hokB* expression, a subpopulation of cells has a depolarized membrane, indicative of pore formation, but does not stain with PI. This indicates that HokB pores can differ in size, with small pores affecting the membrane potential while larger pores are also permeable to PI. Several lines of evidence suggest that the formation of these differently sized HokB pores depends on the membrane potential of the bacterial cell. First, the I-V curve shows nonlinear behavior, indicating that cells with a strongly polarized membrane develop mature HokB pores. Second, PI+ cells have high metabolic activity, which may result from a previously elevated membrane potential that was disseminated by preceding HokB-induced membrane depolarization (33, 38). Accordingly, we hypothesize that HokB induces persistence in cells with a high membrane potential by the formation of mature pores, as shown in *vitro*. Although intermediate pores will cause a decrease in the cellular energy ratio, the effects will be less drastic and will not result in persistence (Fig. 3). Our finding that *hokB*-induced persister cells have high metabolic activity, which allows them to quickly resume growth after removal of the antibiotic, corroborates this model. The link between high metabolic activity and the induction of persistence in stationary phase supports the work of others, who have linked persistence in stationary phase with high metabolic activity (39, 40).

In this work, we unraveled the mode of action of the HokB peptide. We demonstrated that the pore-forming activity of HokB is essential for persistence and results in ATP efflux. Both *in vitro* and *in vivo* data indicate that HokB pore size is controlled by the membrane potential. This way, persistence is induced in metabolically active cells, allowing the HokB-induced persister cells to resume growth quickly after antibiotic removal. The ability of PEG 1000 to reduce the persister fraction upon expression of *hokB* raises the possibility of reducing the number of persister cells induced by type I toxins by using differently sized channel blockers.

## MATERIALS AND METHODS

### Bacterial strains, growth conditions, and plasmids.

E. coli strain BW25113 was used for localization experiments and the persistence assay for the localization constructs (see Fig. S3E in the supplemental material) (41). Other experiments were performed with E. coli TOP10 (Invitrogen, USA). Bacteria were grown in lysogeny broth (LB) at 37°C, and liquid cultures were incubated with orbital shaking (200 rpm).

Construction of pBAD/*Myc*-His A-*mCherry* and pBAD/*Myc*-His A-*PAmCherry* was performed by PCR amplification of *mCherry* or *PAmCherry* from pRSET-B-*mCherry* (42) or pRSET-B-*PAmCherry* (43) with primers CACCGGTACCGTGAGCAAGGGCGAG and ACTGAAGCTTTTACTTGTACAGCTCGTCCA, after which PCR products were digested with KpnI and HindIII. Digested fragments were cloned into pBAD/*Myc*-His A (Invitrogen, USA).

For the construction of pBAD/*Myc*-His A-*mCherry*-*hokB* and pBAD/*Myc*-His A-*PAmCherry*-*hokB*, PCR amplification of *hokB* from pBAD/His A-*hokB* (9) with primers CACCGGTACCGGATCAGGTTCCGGCTCTAAGCACAACCCTCTGG and ATCGAAGCTTTTACCTGGACGTGCA was performed. The PCR product was digested with KpnI and HindIII and cloned into pBAD/*Myc*-His A-*mCherry* or pBAD/*Myc*-His A-*PAmCherry*.

pBAD33-*hokB* was constructed by PCR amplification of *hokB* from pBAD/His A-*hokB* (9) with primers CACCGAGCTCGGAGGAATTAACCATGAAGCACAACCCTCTGGTG and GATCGGTACCGGGATCTCACACTGTACGGC. The PCR product was digested with SacI and KpnI and cloned into pBAD33 (44).

For the construction of pBAD/*Myc*-His A-*Perceval*, primers CCGAGCTCGAGATCTGCACATATGAAACATCACCATCACCA and GTTCGGGCCCAAGCTTCGCTCGAGTCACAATGCTTCCT were used to amplify *Perceval* from pRSET-B-his7-*Perceval* (Addgene plasmid 20336) (34) with overlapping ends to perform Gibson assembly. To this end, the Gibson assembly cloning kit from New England Biolabs (USA) was used.

### Localization of HokB with fluorescence microscopy.

E. coli BW25113 containing pBAD/Myc-His A-*mCherry* or pBAD/*Myc*-His A-*mCherry-hokB* was grown overnight at 37°C under conditions of continuous shaking (200 rpm). The cells were then diluted 100-fold and induced for 4 h with 0.2% arabinose. After 4 h, the cells were pelleted by centrifugation (3,600 rpm, 5 min) and resuspended in phosphate-buffered saline (PBS) buffer. The cells were spotted onto an agar pad and visualized using a Nikon Eclipse Ti-E inverted microscope with a CFI Plan Apochromat 100× objective with a numerical aperture (NA) of 1.45 and excitation at 580 nm. Data were analyzed using the NIS Elements Analysis 4.4 software (Nikon, Japan).

### Localization of HokB with superresolution fluorescence microscopy.

E. coli BW25113 containing pBAD/Myc-His A-*PAmCherry* or pBAD/*Myc*-His A-*PAmCherry-hokB* was incubated overnight at 37°C with continuous shaking (200 rpm). The cells were then diluted 100-fold in fresh LB medium for 2 h and induced with 0.2% arabinose for 3 h. The cells were pelleted by centrifugation (3,600 rpm, 5 min) and resuspended in 10 mM ISO buffer (45). Fixation of the cells was performed with 4% paraformaldehyde in 10 mM ISO buffer for 15 min at room temperature on poly-l-lysine-coated glass slides. Photoactivated localization microscopy (PALM) was performed on an inverted epifluorescence microscope (IX83; Olympus) with a 60× total internal reflection fluorescence (TIRF) oil immersion objective lens (APON 60XOTIRF, NA 1.49; Olympus, Japan). An electron-multiplying charge-coupled-device (CCD) camera (ImagEM C9100-12; Hamamatsu Photonics, Hamamatsu, Japan) was used to capture images. Hokawo Imaging software V2.5 (Hamamatsu Photonics, Japan) was used for recording. Photoconversion of PAmCherry was carried out with a 405-nm laser where the output power was adjusted for each experiment with a neutral density filter, based on the observed switching rate. A 561-nm laser was used for excitation. Fluorescence emission was collected with a 572-nm long-pass and a 590/40 band-pass filter. Lasers were obtained from Coherent, Inc. (USA), and optical filters were from Chroma (Chroma Technology, USA). Images, typically 5,000 frames per experiment, were acquired with 100-ms exposure times. The PALM raw data were analyzed using Localizer software (46) in combination with Igor Pro (Wavemetrics, USA).

### *In vitro* pore formation of HokB*.*

A planar lipid bilayer was formed by the lipid monolayer opposition technique of Montal and Mueller (47). Membranes were prepared with diphytanoylphosphatidylcholine (DPhPC) or with E. coli polar lipid extract dissolved in pentane, both from Avanti Polar Lipids (USA). Planar lipid experiments were conducted under symmetrical conditions in a buffer containing 150 mM NaCl and 10 mM Tris-HCl (pH 7.5). Holding voltages were applied with a 2-kHz low-pass Bassel filter with a 10-kHz sampling rate to the *trans* chamber. The resulting current was recorded using Ag/AgCl electrodes connected to a patch-clamp amplifier (Axopatch 200B; Axon Instruments) and analyzed with the Clampfit 10 software package (Molecular Devices, USA). Bulk solution conductivity was measured with the WTW Multi 3420 conductivity meter in combination with LR 925/01 electrodes and was determined to be 15.4 mS/cm. Synthetic HokB peptides (MKHNPLVVCLLIICITILTFTLLTRQTLYELRFRDGDKEVAALMACTSR) were ordered from Pepscan (The Netherlands) and New England Peptide (NEP) (UK). The peptides were dissolved in dimethyl sulfoxide (DMSO) to a concentration of 10 mM and stored at 4°C for up to 2 weeks. HokB peptides were added at the *trans* side to a concentration of approximately 0.05 mM. When no insertion was observed, more peptide was added to the chamber. For DPhPC lipids, measurements at +100 mV were used for the calculation of the conductance histogram. When the E. coli lipid extract was used, the activating potential (±100 mV) for that specific pore was used. When the observed ion current was unstable and no clear prevalent current was visible, the trace was excluded for further analysis. All measurements were performed at 25°C. The single-pore size was calculated from the single-pore conductance using the model of Hille (22). This model assumes that the pore is a uniform cylinder with conductance *g* and length *l* (assumed to be 30 Å) of the transmembrane domain in a solution with resistivity *ρ* (measured to be 0.649 Ω). The pore diameter *d* is then given by:

$$d=\frac{\rho g}{\pi }\left(\frac{\pi }{2}+\sqrt{\frac{\pi^{2}}{4}+\frac{4\pi l}{\rho g}}\right)$$

For assessing pore open and closing time while gating, pores were considered at their equilibrium (when no new pores inserted in the bilayer). Pore orientation was assessed as described before (29). Peptides were added to the *trans* side of a newly formed bilayer. After pore formation, mPEG5k-mal (stock 10 mM) was added in a concentration of 0.6 mM, first in the *cis* compartment. After approximately 1 min, the same concentration was added in *trans*. mPEG5k-mal was purchased from Sigma-Aldrich (USA).

### Persistence assay.

For the persistence assay in stationary phase, overnight cultures were diluted 100-fold in 250-ml flasks containing 100 ml LB medium, ampicillin (100 µg ml^−1^), and inducer (0.2% arabinose). For the polyethylene glycol (PEG) experiments, PEG (PEG 200, 400, 600, 1000, or 6000) or ethylene glycol was added to the medium. Cultures were incubated for 16 h. All PEGs were purchased from Sigma-Aldrich (USA). Then, 990 µl of culture was treated with 10 µl of 0.5 mg ml^−1^ ofloxacin, and the control treatment was performed with 10 µl sterile water. After 5-h incubation, samples were serially diluted in 10 mM MgSO_4_ and plated on LB agar plates. For persistence measurements in exponential phase, overnight cultures were diluted 100-fold in 250 ml flasks containing the appropriate additives. Samples were taken after 1, 2, 3, and 4 h of incubation, and 990 µl was treated with 10 µl of 0.5 mg ml^−1^ ofloxacin and incubated for 4 h. Untreated samples were plated immediately. The number of CFU was determined after 48-h incubation at 37°C by plate counts. All statistical calculations were performed on log_10_-transformed data and were used to verify potential differences in persister fractions. Statistical comparisons were based on analysis of variance (ANOVA) with a Dunnett’s *post hoc* test for multiple comparisons.

### Determining the energy ratio using Perceval.

E. coli TOP10 cells containing pBAD/*Myc*-His A-*Perceval* and pBAD33 or pBAD33-*hokB* were grown overnight at 37°C under conditions of continuous shaking (200 rpm). The cells were then diluted 100-fold, induced with 0.2% arabinose, and again incubated overnight (16 h). Because Perceval fluorescence is also influenced by pH, cells were diluted in buffered M63 (pH 7) in combination with 40 mM potassium benzoate and 40 mM methylamine hydrochloride to eliminate the transmembrane pH difference just before the measurement. Fluorescence was measured with a Synergy MX multimode reader (BioTek, USA). The excitation spectrum of Perceval shows a large peak around 490 nm and a smaller peak at 405 nm. ATP and ADP competitively bind with Perceval, but binding of ATP results in an enhancement of the 490-nm excitation and a reduction of the 405-nm peak (34). Thus, the 490/405 nm fluorescence ratio is correlated with the ATP/ADP ratio in the cell. Statistical comparisons were based on ANOVA with a Tukey’s *post hoc* test for multiple comparison.

### Determination of ATP in supernatant.

An overnight culture was diluted and induced in flasks containing 100 ml LB. After 1, 2, 3, and 4 h, 1-ml samples were taken. Samples were kept on ice for 5 min and centrifuged for 10 min at 5,000 rpm. One hundred microliters of supernatant was added to 100 µl BacTiter-Glo reagent (Promega, USA), following the manufacturer’s specifications. After 5 min, bioluminescence was measured for every well. At every time point, the optical density (OD) (595 nm) was measured. To take into account the number of cells, the bioluminescence was divided by the OD.

### Determination of protein concentration in supernatant.

An overnight culture was diluted and induced in flasks containing 100 ml LB. After 1, 2, 3, and 4 h, 1-ml samples were taken. Samples were centrifuged for 10 min at 5,000 rpm. Protein concentrations were determined by the Qubit protein assay kit, in accordance with the manufacturer’s specifications (Invitrogen, USA).

### Measuring metabolic activity, membrane potential, and membrane permeability using flow cytometry.

Cells were grown as described above. The cells were diluted in PBS buffer (pH 7.4) and stained following the manufacturer’s instructions. Data were collected for a minimum of 50,000 cells using a BD influx cell sorter (BD Biosciences, USA). For redox sensor green (RSG) staining and bis-(1,3-dibutylbarbituric acid)trimethine oxonol [DiBAC_4_(3)], fluorescence was measured using a 488-nm laser and a 520-nm filter. For propidium iodide (PI), fluorescence was measured using a 488-nm laser and a 620-nm filter. All stains were purchased from Invitrogen (USA). Data were analyzed using FlowJo V10 (BD Biosciences, USA).

### PEG 1000-FITC: localization and flow cytometry.

Cells were grown as described above. After overnight induction in 100-ml flasks, *E*. *coli* TOP10 cells containing pBAD/HisA, pBAD/HisA-*hokB*, or pBAD/mCherry-*hokB* were washed by centrifugation (5000 rpm, 5 min) and diluted in PBS buffer (pH 7.4). PEG 1000-FITC (PEG 1000 labeled with fluorescein isothiocyanate [FITC]), purchased from Creative PEGWorks (USA), was dissolved in PBS buffer (pH 7.4) at a concentration of 3 mg ml^−1^. The cells were incubated for 30 min in the PEG 1000-FITC solution and then washed twice. After the cells were washed, they were spotted onto an agar pad and visualized using a Nikon Eclipse Ti-E inverted microscope with a CFI Plan Apochromat 100× objective with a numerical aperture (NA) of 1.45. Data were analyzed using the NIS Elements Analysis 4.4 software (Nikon, Japan). Correlation between fluorescent patches was assessed using the chi-square statistical test.

### Determination of single-cell lag time.

Cells were grown and treated with antibiotics as described above for the persistence assay in stationary phase. After treatment, cells were resuspended in LB, and a twofold dilution series was prepared. The cells were grown at 37°C with continuous shaking in a Bioscreen C system. Wells with the highest dilution that still displayed growth were selected for single-cell lag time calculation if the two consecutive wells displayed no growth (48). The Gompertz equation was used to calculate lag time (49). Statistical significances of differences were calculated with ANOVA with a Tukey’s *post hoc* test for multiple comparisons.
