# Supplementary figures and images for: The Persistence-Inducing Toxin HokB Forms Dynamic Pores That Cause ATP Leakage
Source: mBio. 2018 Aug 14;9(4):e00744-18. doi: 10.1128/mBio.00744-18 (PMC6094483; doi:10.1128/mBio.00744-18)

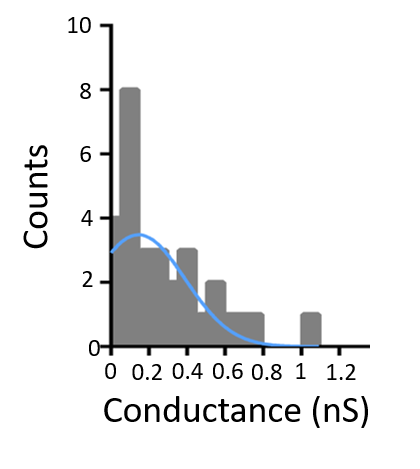

Supplement: FIG S1 [file mbo004184018sf1.tif]

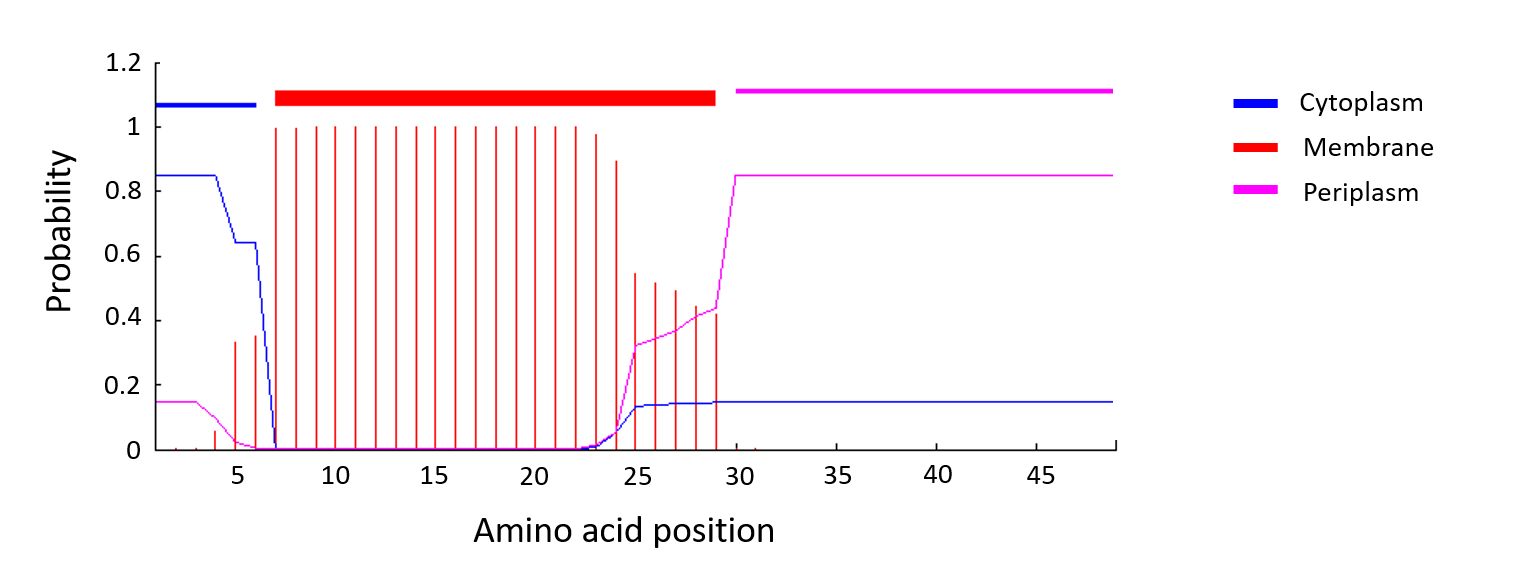

Supplement: FIG S2 [file mbo004184018sf2.tif]

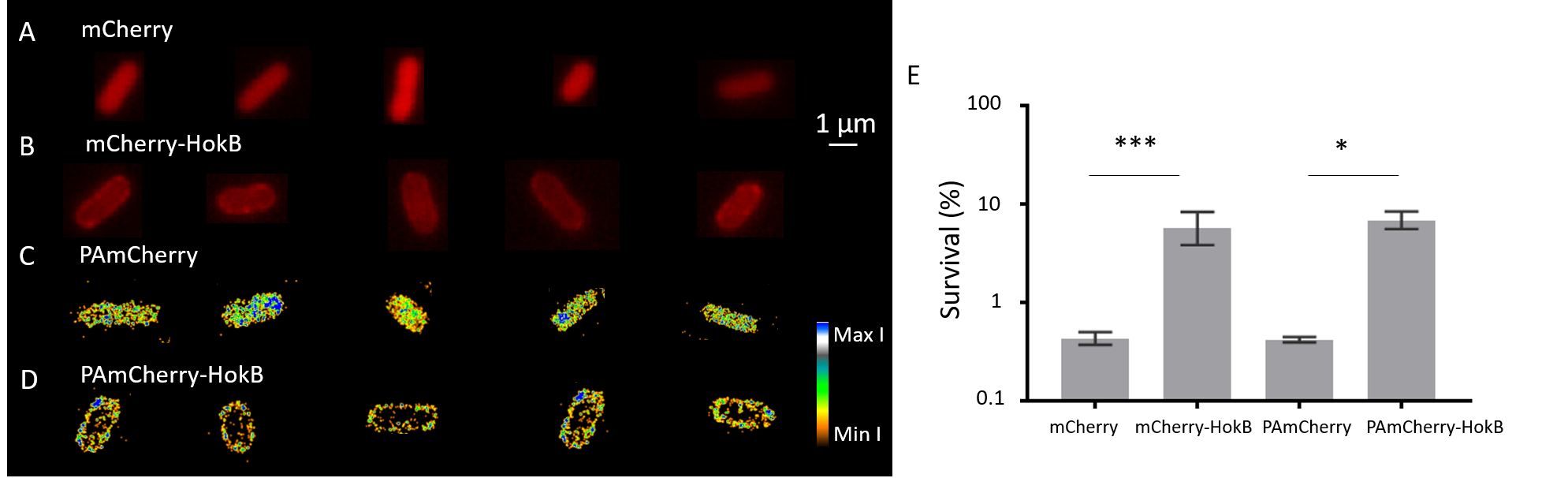

Supplement: FIG S3 [file mbo004184018sf3.tif]

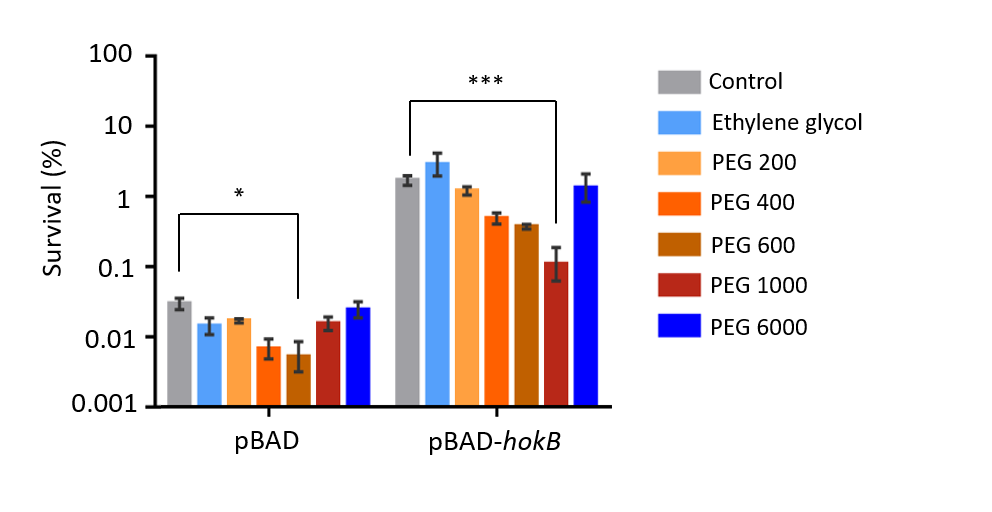

Supplement: FIG S4 [file mbo004184018sf4.tif]

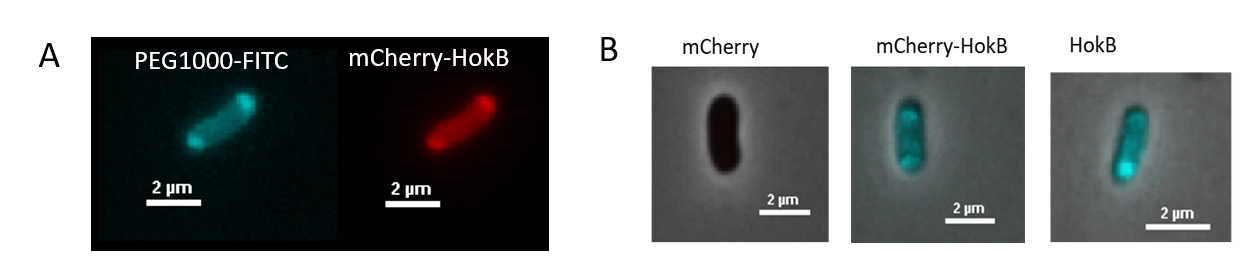

Supplement: FIG S5 [file mbo004184018sf5.tif]
